# Depression among undergraduate medical and engineering students: A comparative study

**DOI:** 10.12669/pjms.36.5.1858

**Published:** 2020

**Authors:** Naveed Ali Siddiqui, Saba Fatima, Fatima Bint Taj, Ayesha Shahid, Zulfiqar Ali Moosa

**Affiliations:** 1 Naveed Ali Siddiqui, MBBS, MPhil, PhD, Associate Professor, Department of Biochemistry,; 2 Saba Fatima, Student, United Medical and Dental College, Karachi, Pakistan; 3 Fatima Bint Taj, Student, United Medical and Dental College, Karachi, Pakistan; 4 Ayesha Shahid, Student, United Medical and Dental College, Karachi, Pakistan; 5 Zulfiqar Ali Moosa, Lecturer, United Medical and Dental College, Karachi, Pakistan

**Keywords:** Depression, Hamilton Depression Scale (HAM-D), Medical students, Engineering students, Student mental health

## Abstract

**Objective:**

To study the depression among medical and engineering students of different medical and engineering colleges in Karachi, Pakistan.

**Methods:**

A comparative cross-sectional study was conducted at different medical and engineering colleges of Karachi from 1^st^ March 2018 till 30^th^ August 2018. Sample size of 362 was calculated by using software SPSS version 22. A close ended, self-administered, modified form of standardized questionnaire was used which comprised of two parts. First part included collection of socio-demographic data, second part had questions for the assessment of depression. Hamilton Depression Scale (HAM-D) was utilized in scoring the depression level in the study subjects.

**Results:**

In engineering and medical colleges 82.87% and 56.9% students were found depressed repeatedly. The result was highly statistically significant. Overall, 109 (30.1%) students were normal, 114 (31.5%) were suffering from mild, 67 (18.5%) moderate, 32 (8.8%) severe and 40 (11.0%) had very severe depression.

**Conclusion:**

In the present study, rate of depression was higher in engineering students as compared to medical students. It is recommended in future that qualitative studies of the causes of depression reducing interventions need to be encouraged in professional program, especially in engineering students.

## INTRODUCTION

Depression is a major illness among psychological disorders which involve people of different age groups and social classes. Anxiety may lead to blood pressure and cardiovascular disease, as well as psychiatric problems like depression.^1^,^2^ Depression can be experienced by a normal human and it is an unavoidable part of existence. 3,4 The study level of Medical and engineering students is notoriously stressful and competitive, requiring long hours of studying, training and practice which ultimately affect physical and psychological well-being of medical and engineering students. Students are overburdened with a massive amount of information, having a limited amount of time to memorize all the information studied, overloaded information creates a feeling of disappointment, inability to handle all the information and increased incidence of errors which ultimately break the stability of the student’s wellness and result in illness.^5^ Coping with depression whilst trying to get through medical and engineering school is not easy.^6^ Consuming excessive caffeinated beverages to be active and alert during the time of studying causes increased levels of adenosine, adrenaline, cortisol and dopamine in the blood, leading to fatigue, depression, behavior changes, heart disease, weight problems, diabetes, and skin diseases.^7^ The perceived depression among medical and engineering students affects not only their academic performance but also their physical and mental health due to which there is a high rate of suicide among them. Engineering students spend most of their time in projects/research work/field work etc and get less time for any activity which helps to relief their stress. Excessive workload and burden of syllabus is a huge obstacle in promoting depression among medical students. That’s why alcohol consumption, drug abuse and suicide rate is high in medical students as compared to other academic programs.^8^

Our objective in this study was to study the depression among medical and engineering students of different medical and engineering colleges in Karachi, Pakistan

## METHODS

A comparative cross-sectional study was organized on randomly selected medical and engineering colleges of Karachi from 1^st^ March 2018 till 30^th^ August 2018. The study was conducted after the approval of United Medical & Dental College, Karachi dated July 26, 2017. The sample size of 362 was calculated by using software SPSS version 22. Sample size was equally divided between two groups i.e. 181 for each. A close ended, self-administered, modified form of standardized questionnaire was used which comprised of three parts. First part included collection of socio-demographic data, second part had questions for the assessment of depression and third part comprised of strategies practiced by students and their respective outcomes. This objective was assessed by Hamilton Depression Rating Scale (HAM-D). The inclusion criteria for this survey included undergraduate medical and engineering students of any gender of Karachi, medical and engineering male and female students of 18-30 years of age. The exclusion criteria for this survey included students having any chronic life threatening disease, students having any psychological disorder, students having family history of psychological disorders, students who refuse to give written consent.

### Statistical Analysis

Data was fed into the computer using Statistical Package for Social Science (SPSS) software program version 22. Data will be presented using descriptive statistic in the form of frequencies and percentage for qualitative variables.

## RESULTS

**Fig.1:**
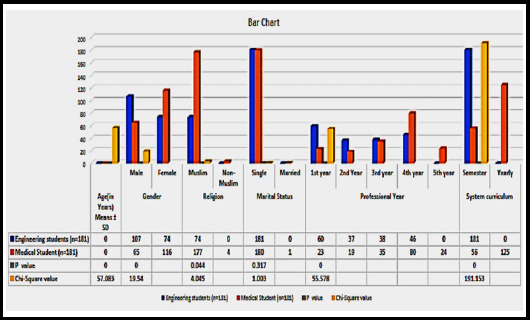
Demographic data of Engineering and Medical students.

Out of total 181 engineering students, 107 were male and 74 were females where as in medical students out of 181, 65 were male and 116 were females. Male students were dominated in engineering while females in medical. In medical 180 students were single while 01 reported as married, on the other hand in engineering all 181 students were single. Muslims dominated in both; medicine (177) and engineering (181). In medical college 23,19,35,80 and 24 students were of 1^st^, 2nd, 3rd, 4^th^ and final professional year respectively. In engineering college 60, 37, 38 and 46 students were of 1^st^, 2^nd^, 3^rd^ and 4^th^ professional year respectively. One hundred twenty five medical students had yearly system curriculum and 56 had semester system while all 181 engineering students had semester system curriculum. The mean age for medical students was 21.81±2.04 and for engineering 20.40±1.523. In medical 43 students were of age ranged between 17-20 years, 130 between 21-24 years and 8 were above 25 years. In engineering 102 were of age ranged between 17-20 years, 79 between 21-24 years. Significant difference was noticed in age groups of respective fields (Fig.1).

**Fig.2:**
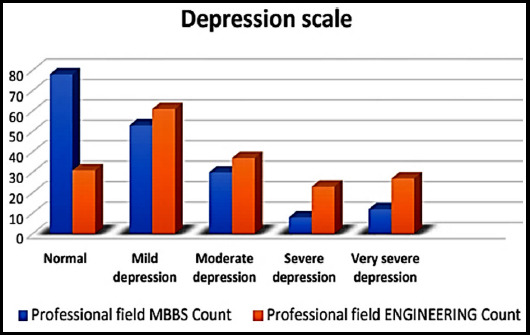
Comparison of level of depression among medical and engineering students.

Overall, 109 (30.1%) students were normal, 114 (31.5%) were suffering from mild, 67 (18.5%) moderate, 32 (8.8%) severe and 40 (11.0%) had very severe depression. Among 181 medical students, 78 (43.1%) were normal, 53 (29.3%) presented with mild depression, 30 (16.6%) had moderate depression, 8 (4.4%) had severe and 12 (6.6%) were found to be severely depressed. Among 181 engineering students, 31 (17.1%) were normal. 61 (33.7%) presented with mild depression, 37 (20.4%) had moderate depression, 24 (13.3%) had severe and 28 (15.5%) were found to be severely depressed (Table-I, Fig.2). The result was highly statistical significant (p-value < 0.05).

**Table-I:** Comparison of level of depression among medical and engineering students (Based on Hamilton Depression Scale).

Depression Scale	Medical Students		Engineering Students		P-value

	Count	Percentage	Count	Percentage	
Normal	78	43.1%	31	17.1%	0.0001*
Mild	53	29.3%	61	33.7%	
Moderate	30	16.6%	37	20.4%	
Severe	8	4.4%	24	13.3%	
Very Severe	12	6.6%	28	15.5%	

TOTAL	181	100.0%	181	100.0%

* P-value significant <0.05.

## DISCUSSION

In India 62% of medical students, 36% of engineering students were witnessing depression.^9^,^10^ According to past studies conducted in this area, the damaging effects of depression and anxiety in the nervous system not only may cause disruption in the fields of education, but also may have some other consequences like cardiovascular disease and cancer.^11^ Studies done at two large universities in Ankara, Turkey, showed that the prevalence of depression among medical and engineering students was 13.8% and 16.4%, respectively.^12^-^14^ Comparatively, the prevalence of depression among engineering students was found to be much higher, with approximately 73.8% of students suffering from depression.^15^ Depression among medical and engineering students is most common as compared to other professional programs. There has not been a single comparative study of depression among medical and engineering students in Karachi, Sindh.

There is high rate of competition in medical and engineering colleges for admission. Students with the high IQ and high scores are selected for the medical and engineering colleges. They have to spend many hours in their studies with less social interaction. Various studies conducted in different localities of Pakistan over the past 10 years give prevalence values of anxiety and depression ranging from 22% to as high as 60% in a given population.^16^ Recent study in Egypt reported that as high as 71 % of university students were having mild depression and around 38% with moderate level of depression. In India 62% of medical students, 86% of engineering students were suffering from depression.^17^ There is an increase rate of competition in medical and engineering studies. It is also found that high level of favoritism leads to the low self-esteem of students in all courses due to which hidden qualities of shy students are never explored which remains hidden throughout the life.

In this study only 109 were normal out of 362 students, 114 had mild depression, 67 were moderate, 32 were severe and 40 had very severe depression. In the present study, 82.87% (181) engineering students were found to be depressed as compared to medical students who exhibited 56.9% (181) which showed significant difference between two groups. In another study which was conducted in India April 2015 by Dr. Maser Khan *et al.*, 62% of medical students were depressed as compared to 36% of engineering students. This showed the higher rates of depression of medical students.^12^ According to the study conducted in India 2016 by Indoo Singh, the rates of anxiety was higher in medical students as compared to engineering students.^6^ In another study conducted in Lahore Pakistan 2016 by Fatima *et al.*, the ratio of depression in engineering students was increased as compared to medical students.^18^,^19^ In this study we observed that depression was more common in female medical students and male engineering students. Depression among first year and second year students was high in engineering but in medical student’s depression was high in third and fourth year. The appearance of depression was decreased in the final year of medical students and was increased in the final year of engineering students.

## CONCLUSION

Depression was common in both medical and engineering students, but it was more prevalent in engineering students as compared to the medical students. Therefore, it is advised to start new programs for the welfare of medical and engineering students and counseling should be initiated by the institution. This study was done in selected private medical and engineering colleges of Karachi on a small scale, more such studies should also be done in different cities of Pakistan so that awareness should be provided properly.

We suggest creating support groups with counseling facilities within medical and engineering colleges in Pakistan. However more advance studies using other scoring tools for depression to explore these findings in Pakistan are needed.

### Author`s Contribution

**NAS** conceived, designed and did statistical analysis & editing of manuscript.

**SF, FBT, AS and ZAM** did data collection, manuscript writing and review of manuscript.

**NAS** is responsible and accountable for the accuracy or integrity of the study.
